# Functional genomic analysis of bile salt resistance in *Enterococcus faecium*

**DOI:** 10.1186/1471-2164-14-299

**Published:** 2013-05-03

**Authors:** Xinglin Zhang, Damien Bierschenk, Janetta Top, Iacovos Anastasiou, Marc JM Bonten, Rob JL Willems, Willem van Schaik

**Affiliations:** 1 Department of Medical Microbiology, University Medical Center Utrecht, Heidelberglaan 100; Room G04.527, Utrecht, 3584 CX, The Netherlands

**Keywords:** *Enterococcus faecium*, Bile resistance, Transposon mutant library, Transcriptome

## Abstract

**Background:**

*Enterococcus faecium* is a Gram-positive commensal bacterium of the mammalian intestinal tract. In the last two decades it has also emerged as a multi-resistant nosocomial pathogen. In order to survive in and colonize the human intestinal tract *E. faecium* must resist the deleterious actions of bile. The molecular mechanisms exploited by this bacterium to tolerate bile are as yet unexplored.

**Results:**

In this study we used a high-throughput quantitative screening approach of transposon mutant library, termed Microarray-based Transposon Mapping (M-TraM), to identify the genetic determinants required for resistance to bile salts in *E. faecium* E1162. The gene *gltK*, which is predicted to encode a glutamate/aspartate transport system permease protein, was identified by M-TraM to be involved in bile resistance. The role of GltK in bile salt resistance was confirmed by the subsequent observation that the deletion of *gltK* significantly sensitized *E. faecium* E1162 to bile salts. To further characterize the response of *E. faecium* E1162 to bile salts, we performed a transcriptome analysis to identify genes that are regulated by exposure to 0.02% bile salts. Exposure to bile salts resulted in major transcriptional rearrangements, predominantly in genes involved in carbohydrate, nucleotide and coenzyme transport and metabolism.

**Conclusion:**

These findings add to a better understanding of the molecular mechanisms by which *E*. *faecium* responds and resists the antimicrobial action of bile salts.

## Background

*Enterococcus faecium* is a common inhabitant of the intestines of humans and animals and is present in many different natural environments [1,2]. However, during the past two decades *E. faecium* has rapidly emerged as an important multi-drug resistant nosocomial pathogen around the world and is now frequently responsible for hospital-acquired bloodstream, urinary tract and surgical wound infections [3-5]. The establishment of high-level intestinal colonization by enterococci is a crucial step in a process that can finally lead towards nosocomial infections [5].

Enterococci are known as being highly tolerant to hostile environments including high temperature conditions and high salt concentrations [6]. Enterococci are also relatively resistant to chemical disinfectants like chlorine, glutaraldehyde and alcohol [7-9]. In order to survive in and colonize the human intestinal tract, a bacterium must be able to adapt to the stressful conditions that occur in this environment. Bile represents a major challenge to the intestinal microflora. The human liver daily secretes up to one liter of bile which is stored in the gall bladder and exported into the intestine [10]. Bile is a complex mixture composed mainly of bile salts, phospholipids, cholesterol, proteins and bilirubin [11]. Bile salts are amphipathic molecules that act as detergents, aiding in lipid solubilization and digestion, but they also play a role in host defenses, as bile salts have potent antimicrobial properties that can cause damage to the DNA, proteins and membranes of enteric bacteria [12,13]. In both Gram-positive and Gram-negative bacteria the disruption of bile tolerance loci often leads to impaired intestinal survival [14-16], while a mutation resulting in high-level bile resistance of *Escherichia coli* results in a fitness advantage during intestinal colonization [17].

As a successful colonizer of the intestinal tract, *E. faecium* must have developed mechanisms to sense, respond to and tolerate bile during its evolution as a gut commensal. Previously, two genetic loci (*gls33*-*glsB* and *gls20*-*glsB1*) that encode Gls-like proteins in *E. faecalis* and *E. faecium* were identified to be involved in bile resistance and pathogenicity in a mouse peritonitis model [18,19]. *E. faecium* was also possesses bile salt hydrolase activity [20], which is conferred by the protein encoded by the *bsh* gene (accession no. AY260046) [21]. In this study, we performed a genome-wide identification of the genetic loci required for bile salt resistance in *E*. *faecium*, using a high-throughput quantitative screening approach of transposon mutant libraries, termed Microarray-based Transposon Mapping (M-TraM) [22]. We also studied the transcriptional response of *E*. *faecium* to bile salts-induced stress.

## Methods

### Bacterial strains, plasmids and growth conditions

*E. faecium* and *E. coli* strains used in this study are listed in Table 1. The *E. faecium* strain E1162 was used throughout this study. This strain was isolated from a bloodstream infection in France in 1996 and its genome has previously been sequenced [23]. Unless otherwise mentioned, *E. faecium* was grown in brain heart infusion broth (BHI; Oxoid) at 37°C. The *E. coli* strains DH5α (Invitrogen) and EC1000 [24] were grown in Luria-Bertani medium. Where necessary, antibiotics were used at the following concentrations: gentamicin at 300 μg ml^−1^ for *E. faecium* and 25 μg ml^−1^ for *E. coli*, spectinomycin at 300 μg ml^−1^ for *E. faecium* and 100 μg ml^−1^ for *E. coli*. All antibiotics were obtained from Sigma-Aldrich (Saint Louis, MO). Growth of cultures was determined by measuring the optical density at 660 nm (OD_660_).

**Table 1:** Strains and plasmids used in this study

**Strain or plasmid**	**Relevant characteristic(s)**		**Source or reference**
*E****. ****faecium*			
E1162	Clinical isolate (bloodstream infection), isolated in France, 1996		[23]
Δ*gltK*	Markerless deletion mutant of *gltK* gene of E1162		This study
Δ*gspA*	Markerless deletion mutant of *gspA* gene of E1162		This study
Δ*gltK+gltK*	Complementation strain of Δ*gltK*; Δ*gltK* harboring pEF25-*gltK*		This study
Δ*gspA+gspA*	Complementation strain of Δ*gspA*; Δ*gspA* harboring pEF25-*gspA*		This study
*E. coli* strains			
DH5*α*	*E. coli* host strain for routine cloning		Invitrogen
EC1000	MC1000 *glgB*::*repA*; host strain for pWS3 derived vectors		[24]
Plasmids			
pWS3	Gram-positive thermosensitive origin of replication; Spc^r^		[29]
pDEL3a	pWS3 carrying the 5′ and 3′ flanking regions of gene *gltK* for mutant construction		This study
pDEL4a	pWS3 carrying the 5′ and 3′ flanking regions of *gspA* gene cluster for mutant construction		This study
pDEL3b	pDEL3a with a Gen^r^ cassette which was flanked by *lox66*- and *lox71*-sites cloned between the 5′ and 3′ flanking regions		This study
pDEL4b	pDEL4a with a Gen^r^ cassette which was flanked by *lox66*- and *lox71*-sites cloned between the 5′ and 3′ flanking regions		This study
pWS3-Cre	pWS3 derivative expressing Cre in *E. faecium*		[22]
pEF25	Shuttle plasmid pAT18 with spectinomycin resistance cassette cloned in the EcoRI site; Spc^r^ Ery^r^		[30]
pEF25-*gltK*	Complementation plasmid for Δ*gltK*; pEF25 carrying *gltK*		This study
pEF25-*gspA*	Complementation plasmid for Δ*gspA*; pEF25 carrying *gspA*		This study

### Screening for genes involved in bile salt resistance using M-TraM

M-TraM, a high throughput screening technique of transposon mutant libraries has previously been described in detail [22]. Here we use this technique to perform a genome-wide identification of genes involved in bile salt resistance in *E. faecium*. Briefly, aliquots containing approximately 10^7^ colony-forming units (CFU) from the mutant pool were used to inoculate 20 ml of BHI broth or BHI broth supplemented with 0.02% bile salts (sodium cholate:sodium deoxycholate 1:1, Sigma-Aldrich). Cells were grown at 37°C for 20 hours, after which 1 ml of the cultures were spun down and used for the extraction of genomic DNA, which was then further processed as described previously [22]. Statistical differences in hybridization signals between the conditions were analyzed using Cyber-T [25] (http://cybert.microarray.ics.uci.edu/). Probes exhibiting a Bayesian P-value <0.005 were deemed statistically significant. A gene of which at least two identical probes (two different probes per gene were spotted in duplicate on the microarray [22]) passed this threshold were classified as significantly selected during exposure to bile salts. In an addition, genes which were selected between 0.5- and 2-fold were deemed biologically insignificant and were filtered out. This experiment was performed with four biological replicates.

The microarray data generated in the M-TraM screening have been deposited in the ArrayExpress database (http://www.ebi.ac.uk/arrayexpress) under accession number E-MEXP-3797.

### Transcriptome profiling

*E. faecium* E1162 was grown in 3 ml BHI broth at 37°C for 18 hours. Cultures were then diluted 100 fold in 20 ml of prewarmed BHI broth (in a 50-ml Falcon tube) and grown until OD_660_ 0.3. Two ml aliquots of the cultures were centrifuged for 12 seconds at 16900 *g* at room temperature, and pellets were flash frozen in liquid N_2_ prior to RNA extraction. This sample served as the negative control (t = 0 min) prior to the addition of bile salts. Bile salts (final concentration 0.02%, w/v) were added into the remaining 18 ml of culture. After 5 and 15 minutes of incubation at 37°C, 2 ml aliquots of the cultures were centrifuged and flash frozen as described above. RNA isolation, cDNA synthesis and hybridization were performed as described in our previous work [22]. In this experiment, the expression of genes at t = 5 min and t = 15 min were compared to t = 0 min. Analysis for statistical significance was performed using the Web-based VAMPIRE microarray suite (http://sasquatch.ucsd.edu/vampire/) as described previously [26,27]. A gene of which all four probes (two different probes were spotted in duplicate on the microarray [22]) were identified as differentially expressed with a false discovery rate <0.001, were classified as significantly different between samples. Genes with an expression ratio between 0.5- and 2-fold were deemed biologically insignificant and were filtered out. This experiment was performed with two biological replicates.

The microarray data generated in the transcriptome analysis have been deposited in the ArrayExpress database (http://www.ebi.ac.uk/arrayexpress) under accession number E-MEXP-3796.

### Construction of markerless deletion mutants and *in trans* complementation

Markerless gene deletion mutants in the *gltK* gene (locustag: EfmE1162_1760) and the *gspA* gene (locustag: EfmE1162_1186) were created via the Cre-*lox* recombination system as previously described [22,28]. Briefly, the 5′ and 3′ flanking regions (approximately 500 bp each) of the target genes were PCR amplified with the primers in Table 2. The two flanking regions were then fused together by fusion PCR (generating an EcoRI site between both fragments) and cloned into pWS3 [29], resulting in pDEL3a and pDEL4a (plasmids used or generated in this study are listed in Table 1). Then, a gentamicin-resistance cassette which was flanked by *lox66*- and *lox71*-sites [22] was cloned into the EcoRI site that was generated between the 5′ and 3′ flanking regions in pDEL3a and pDEL4a, respectively. The resulting plasmids pDEL3b and pDEL4b were then electrotransformed into *E. faecium* E1162. Marked deletion mutants were obtained by growing the gentamicin-resistant transformants as described previously [22]. The plasmid pWS3-Cre [22], carrying a gene encoding Cre recombinase, was introduced into the marked mutant by electroporation. Further culturing for the removal of the gentamicin resistance cassette and subsequent loss of pWS-Cre was performed as described previously [22]. Excision of the gentamicin resistance cassette and loss of pWS3-Cre was verified by PCR using primers listed in Table 2.

**Table 2:** Primers used in this study

** Primer**	** Sequence ^a^**
delete_XmaI_gltK_up_F	5′-CCCCCCGGGCCAAGCAGGTACGATTGGAT-3′
delete_EcoRI_gltK_up_R	5′-AACCGGAAAGCAGAGAATTCTCGAAAACAATGAAACTTCAACA-3′
delete_EcoRI_gltK_dn_F	5′-TCGAGAATTCTCTGCTTTCCGGTTACTTGG-3′
delete_XhoI_gltK_dn_R	5′-CCGCTCGAGGGAAGGATCACACCGATGAC-3′
check_gltK_up	5′-CGGAACGTTAATGGCAATCT-3′
check_gltK_dn	5′-CCGTACCAATCGTACCGATAA-3′
delete_XmaI_gspA_up_F	5′-CCCCCCGGGCCTCCTTTTTGGACTTTCTCG-3′
delete_EcoRI_gspA_up_R	5′-ACCACATTTAGCTGCAGAATTCGACGGCTTTCCGTTGTGTAG-3′
delete_EcoRI_gspA_dn_F	5′-CGAATTCTGCAGCTAAATGTGGTACGAA-3′
delete_XhoI_gspA_dn_R	5′-CCGCTCGAGGCCAAGTGAAAGCTTTGGAA-3′
check_gspA_up	5′-GCTCGAATTCTTCGATTGCT-3′
check_gspA_dn	5′-TGATGAGCCGTTAAATGGAA-3′
complement_BamHI_gltK_F	5′-ACGGGATCCTTTTAGCAATCGTAGCTGGTTT-3′
complement_XhoI_gltK_R	5′-ACCGCTCGAGCGTGAATTTTCAAGTGCTC-3′
complement_BamHI_gspA_F	5′-ACGGGATCCTGAAAAACCTTCGATCGTTCA-3′
complement_XhoI_gspA_R	5′-ACCGCTCGAGTCCATTCCTACTCCCCCTCT-3′
pAT392_EcoRI_lox66_genta_F	5′-AGGGAATTCTACCGTTCGTATAGCATACATTATACGAAGTTATG ATAAACCCAGCGAACCATTTGAGG-3′
pAT392_EcoRI_lox71_genta_R	5′-CTCCGAATTCTACCGTTCGTATAATGTATGCTATACGAAGTTATT CAATCTTTATAAGTCCTTTTATAA-3′

^a^ Restriction sites are underlined.

*In trans* complemented strains of *gltK* and *gspA* gene deletion mutants were generated as described previously [22,30]. The *gltK* and *gspA* genes were PCR amplified, respectively, from the genomic DNA of E1162 using the primers listed in Table 2. The PCR products were cloned into the shuttle vector pEF25 [30]. The resulting plasmids, pEF25-*gltK* and pEF25-*gspA*, were introduced into the corresponding mutant strains by electroporation as described above.

### Determination of growth curves

A BioScreen C instrument (Oy Growth Curves AB, Helsinki, Finland) was used to determine the effects of bile salts on bacterial growth. Wild type, mutants and the *in trans* complemented strains were grown overnight in BHI (containing appropriate antibiotics for the *in trans* complemented strains). Cells were inoculated at an initial OD_660_ of 0.0025 into 300 μl BHI and BHI with 0.02%, 0.04%, 0.08% and 0.16% of bile salts, respectively. The cultures were incubated in the Bioscreen C system at 37°C with continuous shaking, and absorbance of 600 nm (A_600_) was recorded every 15 min for 12 hours. Each experiment was performed in triplicate.

### Bile salt adaptation and challenge assays

To compare the sensitivity to bile salts of the parental strain E1162, the mutant strains and *in trans* complemented strains, overnight cultures were diluted 100 fold in fresh BHI and grown to OD_660_ 0.3. One ml of the cell cultures were harvested by centrifugation at 12500 *g* for 1 minute and adapted to bile salts by resuspending the cells in BHI containing sub-lethal levels of bile salts (0.02%) or in BHI without any additions. After a 15-minute adaptation period, viable counts were determined by serial dilution and plating on BHI agar plates (time point 0). Adapted and non-adapted cells were spun down as described above and resuspended in BHI containing 0.3% bile salts, which corresponds to a concentration that is commonly reached in the human small intestine after ingestion of a meal [31]. After 5, 30 and 60 minutes of incubation at 37°C, aliquots of cells were washed with PBS and viable counts were determined following serial dilution and plating on BHI agar plates. The experiment was performed in triplicate and statistical analysis of the data was performed using an unpaired two-tailed Student’s *t*-test.

## Results and discussion

### Identification of genetic determinants involved in bile salt resistance in *E. faecium* by M-TraM

To identify genes that are required for bile salt resistance in *E. faecium* E1162, we grew the pool of mutants in the presence or absence of a sub-lethal concentration (0.02%) of bile salts for 20 hours, and used M-TraM to determine which mutants were less resistant to bile salts and therefore are selectively lost during culturing in the presence of bile salts. Seventy-five genes belonging to a variety of functional categories were identified to be involved in bile resistance (Additional file 1 and 2). A single gene, *gltK* (locus tag EfmE1162_1760), encoding a putative glutamate/aspartate transport system permease protein, was identified by M-TraM with the highest fold change (11.5 fold, which was notably higher than the other identified genes), indicating that this gene may contribute considerably to bile resistance in *E. faecium*. Consequently, we decided to further study the function of this gene in bile resistance (further described below). We were unable to find previous studies that linked GltK and its homologues in other microorganisms to bile resistance. BLAST analysis showed that GltK is conserved (with amino acid identities >97%) in all of the 69 *E. faecium* genomes available (on 30 October 2012) at NCBI Genomes, indicating that the *gltK* gene is part of the *E. faecium* core genome. Another gene that was identified as contributing to bile resistance by M-TraM analysis was a gene (locus tag: EfmE1162_2043) encoding a putative cardiolipin synthetase, which functions as an enzyme in phospholipid metabolism and is involved in enterococcal daptomycin resistance [32,33]. It possibly acts by protecting the cells from membrane-associated damage induced by bile. In *E. faecalis*, the *sagA* gene was previously shown to be important in maintaining cell wall integrity and resistance to bile [34]. The *E. faecium* homolog (locus tag: EfmE1162_2437) of the *sagA* gene was also identified by M-TraM as potentially contributing to bile resistance. The *bsh* gene (locus tag: EfmE1162_2656) which encodes a bile salt hydrolase (BSH) [21] is conserved in all the 69 publicly available *E. faecium* genomes, including E1162. However this gene was not identified by M-TraM screening, presumably because BSH does not provide protection despite its predicted activity in the hydrolysis of bile salts. It is also possible that in the M-TraM screening, during which many different transposon insertion mutants are pooled together, the minor proportion of BSH-deficient mutants could be compensated by the extracellular bile salt hydrolase activity that is produced by cells that carry other mutations. We did not identify the two Gls-like protein-encoding loci which were shown to be involved in bile resistance in a previous study [19]. However, single deletions of either locus only resulted in a minor effect on bile salt resistance possibly due to mutual compensation of the two loci [19], which may also explain why we did not identify these loci in the M-TraM screening, as the mutant library only contains mutants that are inactivated in a single locus by transposon insertion [22].

### Transcriptional responses of *E. faecium* to bile salt-induced stress

A microarray-based transcriptome analysis was used to identify genes that are regulated by exposure to bile salts. Compared to the untreated control, 214 (175 up-regulated and 39 down-regulated) and 190 (119 up-regulated and 71 down-regulated) genes were identified to be differentially expressed at 5 min and 15 min incubation with bile salts, respectively (Additional files 2 and 3). The data of the transcriptional analyses at the two different time points (t = 5 min and t = 15 min) exhibited a correlation (R^2^ of log_2_-transformed values) of 0.44 with each other (Additional file 4A). However the transcriptome data are completely uncorrlated with the M-TraM analysis (R^2^ of log_2_-transformed values ≤ 0.001) (Additional file 4BC), which is consistent with previous observations that gene expression poorly correlates with mutant fitness measurements [22,35].

**Figure 1:**
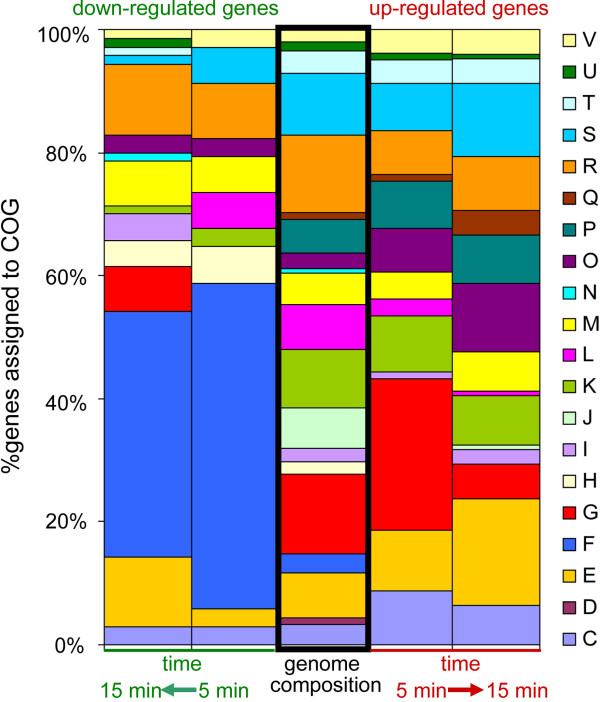
**COG classification of differentially expressed genes during exposure to bile salts.** The down-regulated (left two columns) and up-regulated (right two columns) genes during bile treatment were grouped by COG functional categories, respectively, and the percentage abundance of each group was compared to abundance of each COG in the E1162 genome. The one-letter codes represent the following COG functional categories: C: energy production and conversion; D: cell cycle control, cell division, chromosome partitioning; E: amino acid transport and metabolism; F: nucleotide transport and metabolism; G: carbohydrate transport and metabolism; H: coenzyme transport and metabolism; I: lipid transport and metabolism; J: translation, ribosomal structure and biogenesis; K: transcription; L: replication, recombination and repair; M: cell wall/membrane/envelope biogenesis; N: cell motility; O: posttranslational modification, protein turnover; chaperones; P: inorganic ion transport and metabolism; Q: secondary metabolites biosynthesis, transport and catabolism; R: general function prediction only; S: function unknown; T: signal transduction mechanisms; U: intracellular trafficking, secretion, and vesicular transport; V: defense mechanisms.

Genes identified at either time points were grouped by COG functional categories and the percentage abundance of each group was compared to the overall COG-based composition of the E1162 genome [23] (Figure 1 and Additional file 5). Genes in COG categories F (nucleotide transport and metabolism) and H (coenzyme transport and metabolism) were overrepresented among the down-regulated genes during exposure to bile salts, and no genes from these categories exhibited up-regulated expression during bile salt exposure, indicating that the decreased expression of genes in these two functional categories is a major transcriptional response of *E. faecium* to bile salts. Among the up-regulated genes, genes in COG category G (carbohydrate transport and metabolism) were overrepresented at 5 min after exposure to bile salts. These included genes that are predicted to encode proteins involved in the utilization of a variety of sugars including maltose, maltodextrin, cellobiose, galactose, fructose, mannose and lactose. No genes from COG category G were down-regulated after 5 min. This observation indicates that bile salts positively impact on expression of genes involved in carbohydrate transport and metabolism immediately after exposure to bile salts, but this response becomes less prominent after longer periods of time. We also found a number of categories that were enriched in up-regulated genes, including C (energy production and conversion), E (amino acid transport and metabolism), O (posttranslational modification, protein turnover; chaperones) and Q (secondary metabolites biosynthesis, transport and catabolism). These data suggest an involvement of these functional categories in the *E. faecium* response to bile salts.

We further focused on a gene EfmE1162_1186 (*gspA*) which is predicted to encode a general stress protein A. This gene was identified by both transcriptome analysis (4.6 and 47.0 fold up-regulated at 5 min and 15 min of bile salts treatment, respectively) and M-TraM (2.8 fold less signal in bile-exposed library compared to the control). GspA is also highly conserved (with amino acid identities >98%) in 66 of the 69 *E. faecium* genomes. We observed that both of the two Gls-like protein-encoding loci (EfmE1162_1192-EfmE1162_1193 and EfmE1162_1201-EfmE1162_1202) were induced over eight-fold during exposure to bile salts, although they were not identified by M-TraM screening. However, the *bsh* gene was not identified to be differentially expressed in BHI with bile salts, indicating that the expression of this gene is not regulated by bile salts despite its predicted role in bile salt hydrolysis.

The transcriptional responses of *E. faecalis* to bovine bile has been investigated in a previous study [36]. A striking common finding of this study and our work is that a large gene cluster (locus tags EfmE1162_0724-EfmE1162_0731 in *E. faecium* E1162 and EF1492-EF1500 in *E. faecalis* V583), which putatively encodes a V-type ATPase, exhibits upregulated expression during exposure to bile salts. V-type ATPases are membrane proteins that function as proton- or sodium ion pumps that build up ion gradients at the expense of ATP [37]. Induction of this gene cluster suggested that *E. faecium* may generate a proton gradient to respond to bile mediated stress. The link between bile mediated stress and maintenance of the proton motive force (PMF) was previously demonstrated in the Gram-positive bacteria *Lactobacillus plantarum*[38], *Bifidobacterium longum*[39] and *B. animalis*[40]. Bile salts can induce DNA damage in bacteria, and consequently DNA mismatch repair proteins are important for bacterial bile resistance [12,41,42]. In this study we identified a gene (locus tag: EfmE1162_1335), encoding the DNA mismatch repair protein MutS, that was higher expressed (23.0 fold at 5 min and 9.5 fold at 15 min) after addition of bile salts to the culture medium.

### Effect of bile salts on growth of *E. faecium* E1162 wild-type and *gltK* and *gspA* mutants

**Figure 2:**
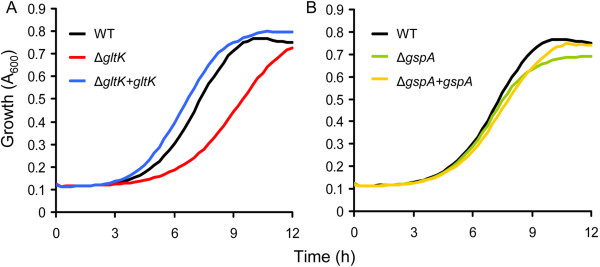
**Growth of *E. faecium *in BHI with 0.08% bile salts.** Overnight cultures of *E. faecium* strains were inoculated at an initial cell density of OD_660_ 0.0025 in BHI with 0.08% bile salts. Growth curves of wild-type E1162, the two mutants (panel **A**: Δ*gltK*, and panel **B**: Δ*gspA*) and *in trans* complemented strains are shown. Growth curves are the means of three independent experiments.

To determine the role of GltK and GspA in bile salt resistance, markerless deletion mutants in *gltK* and *gspA* were constructed in *E. faecium* E1162, and the mutants were complemented *in trans*. The growth of *E. faecium* E1162 wild type (WT), the isogenic mutants and the complemented strains in BHI and BHI supplemented with bile salts were determined. In the absence of bile salts the wild-type strain and its isogenic mutants grew identically (Additional file 6). When these strains were grown in BHI with 0.02%, 0.04%, 0.08%, 0.16% and 0.32% bile salts, the growth rate of the Δ*gltK* mutant decreased compared to WT and this difference was most notable in BHI with 0.08% bile salts (Additional file 6 and Figure 2). The growth rate of the Δ*gltK* mutant could be restored to WT levels upon *in trans* complementation (Figure 2), indicating that GltK contributes to bile resistance of *E. faecium*. Only very minor effects on growth rate and optical density in stationary phase were observed upon deletion of *gspA,* indicating that this gene has an insignificant role in bile resistance of *E. faecium* despite its highly induced expression upon exposure to bile salts. In addition, the sensitivity of both mutants to other stresses, including different pHs, oxidative and osmotic stress, were examined and no significant difference between the mutants and wild-type strain was detected (data not shown).

### Bile salt adaptation and challenge assays

**Figure 3:**
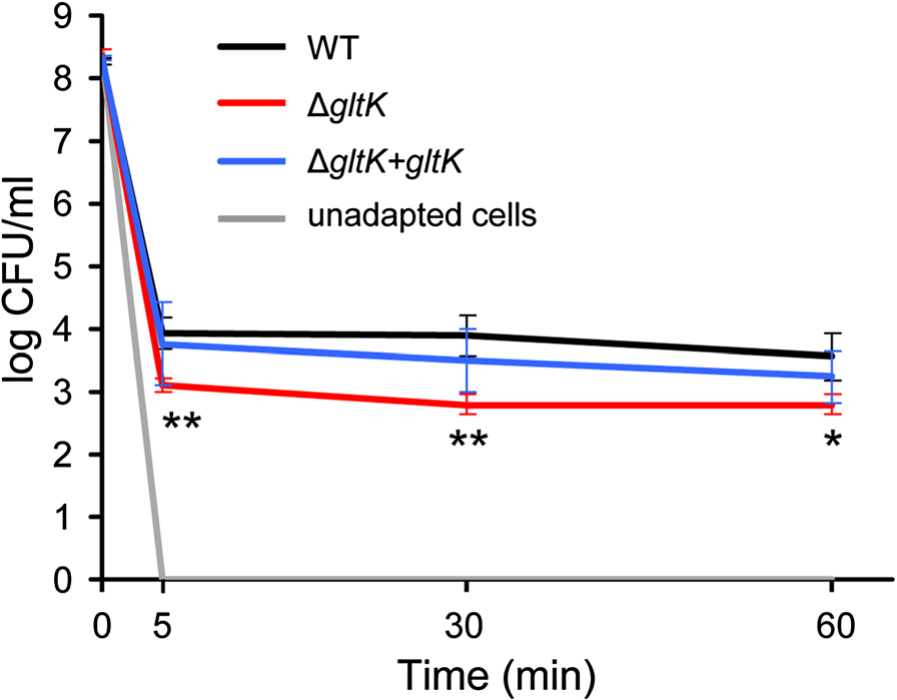
**Survival of *E. faecium *cells following exposure to 0.3% bile salts with and without pre-adaptation.** Exponential-phase cells of the E1162 wild type, the Δ*gltK* mutant and its *in trans* complemented strain Δ*gltK+gltK* were adapted to 0.02% bile salts for 15 min or were left unadapted, and then challenged with 0.3% bile salts. Viable cells were determined immediately before exposure to 0.3% bile salts (t = 0 min) and after 5, 30 and 60 min of challenge. The grey line represents unadapted cultures of these strains, in which viable counts dropped to undetectable levels (<50 CFU/ml) after being challenged by bile salts. Bars represent the standard deviation of the mean of three independent experiments. Asterisks represent significant differences (*: *P*<0.05, **: *P*<0.01, as determined by an unpaired two-tailed Student’s *t*-test) between the Δ*gltK* mutant and the wild type strain.

Exponential-phase cells of E1162 wild-type, the Δ*gltK* mutant and the *in trans* complemented strain were adapted to 0.02% bile salts for 15 min or were left unadapted, and then challenged with 0.3% bile salts (Figure 3). Viable counts for the unadapted cells dropped below the detection limit (<50 CFU/ml) after the challenge, indicating that these cells were sensitive to this high concentration of bile salts. In contrast, the adapted cells were more tolerant to 0.3% bile salts, with 10^4^ CFU/ml surviving after 5 min of being exposed to bile salts and no significant further killing occurring during the remainder of the 1-hour experiment. These results showed that adaptation to low levels of bile salts provided *E. faecium* substantial protection to levels of bile salts that are lethal to non-adapted cells. The deletion of *gltK* reduced the protection provided by the adaptation to a sub-lethal concentration of bile salts, leading to an approximately 1-log lower survival of pre-adapted *gltK* cells than the survival of the wild type E1162 cells. Survival of the *in trans* complemented strain upon pre-adaptation to bile salts was similar to that of the wild type. The Δ*gspA* mutant was also included in this assay, but no significant difference was observed compared to wild-type E1162 (data not shown), again indicating that *gspA* was not required for bile resistance although its expression was highly induced by bile salts.

Our data suggest that bile salt-regulated genes do not necessarily contribute to bile resistance. Previous studies indicated that the protective adaptation to bile salts mainly results from changes in membrane composition and architecture that are independent of *de novo* protein synthesis [43,44]. Flahaut *et al*. showed that a 5 second-adaptation of *E. faecalis* to low level bile salts could provide substantial protection against challenge with lethal bile salt concentrations, and the addition of chloramphenicol during the adaptation period did not prevent development of acquired tolerance [44]. A similar result was also observed in *L. monocytogenes*[43]. However, the bile salt-regulated genes, rather than directly contributing to bile resistance, could be involved in other functions including virulence and carbohydrate metabolism [10]. It has previously been established in *Salmonella*[45,46] and *Vibrio*[47-49] that bile can be used as an environmental cue to influence the regulation genes involved in intestinal colonization and virulence. We identified many genes involved in carbohydrate metabolism that exhibited upregulated expression upon exposure to bile salts, e.g. a gene cluster (locus tags: EfmE1162_1484 - EfmE1162_1489) putatively involved in maltose utilization (Zhang *et al*., unpublished work). This may suggest that *E. faecium* senses bile as an environmental signal indicating that it has entered the host gut, leading to a rapid adjustment of the cell’s carbohydrate metabolism.

## Conclusions

Responding and being resistant to bile are important features of bacteria that inhabit the gut [10]. In the present work, we have identified a genetic determinant in *E. faecium* that contributes to bile salt resistance, and studied the transcriptional response of *E*. *faecium* to bile salts. These findings add to a better understanding of the molecular mechanisms that lead to bile resistance in *E. faecium*.

## Competing interests

The authors declare that they have no competing interests.

## Authors’ contributions

XZ and WvS designed research. XZ, DB, JT and IA performed experiments. XZ, RJW and WvS analyzed data. XZ, RJW, MJMB and WvS wrote the manuscript. All authors read and approved the final manuscript.

## Supplementary Material

Additional file 1: Table S1 Complete data of gene expression ratios (t = 5 min and t = 15 min versus t = 0 min) and M-TraM analysis of *E. faecium* E1162. Click here for file

Additional file 2: Table S2 Complete data of gene expression ratios (t = 5 min and t = 15 min versus t = 0 min) and M-TraM analysis of *E. faecium* E1162. Click here for file

Additional file 3: Table S3 Expression ratios (t = 5 min and t = 15 min versus t = 0 min) of *E. faecium* E1162 genes that are significantly differentially expressed upon exposure to bile salts. Click here for file

Additional file 4: Figure S1 Comparison of transcriptome analysis (gene expression) and M-TraM analysis (mutant fitness). Each dot represents a gene probe. The axes represent the log_2_-transformed fold-changes in either transcriptome or M-TraM analysis. (A) Transcriptome (t = 15 min) versus transcriptome (t = 5 min). (B) Transcriptome (t = 5 min) versus M-TraM. (C) Transcriptome (t = 15 min) versus M-TraM. (TIFF 376 kb) Click here for file

Additional file 5: Table S4 COG classification of differentially expressed genes during exposure to bile salts. Click here for file

Additional file 6: Figure S2 Growth of *E. faecium* in BHI with different concentrations of bile salts. Overnight cultures of *E. faecium* strains were inoculated at an initial cell density of OD_660_ 0.0025 in BHI or BHI with 0.02%, 0.04%, 0.08%, 0.16% and 0.32% of bile salts. Growth curves of wild-type E1162 and the Δ*gltK* mutant are shown. Click here for file
