# Supplementary figures and images for: Functional genomic analysis of bile salt resistance in Enterococcus faecium
Source: BMC Genomics. 2013 May 3;14:299. doi: 10.1186/1471-2164-14-299 (PMC3653699; doi:10.1186/1471-2164-14-299)

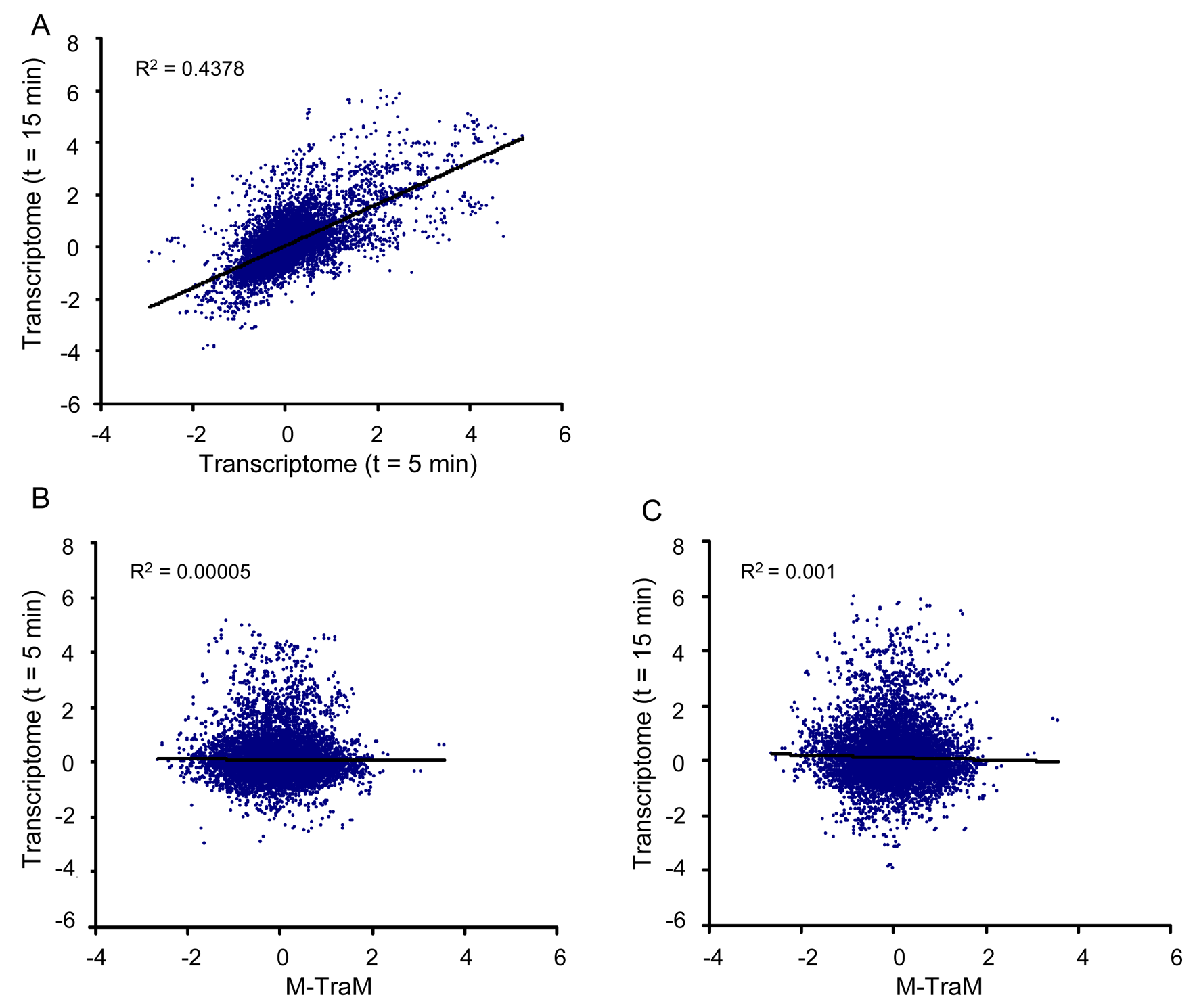

Supplement: Additional file 4: Figure S1 — Comparison of transcriptome analysis (gene expression) and M-TraM analysis (mutant fitness). Each dot represents a gene probe. The axes represent the log2-transformed fold-changes in either transcriptome or M-TraM analysis. (A) Transcriptome (t = 15 min) versus transcriptome (t = 5 min). (B) Transcriptome (t = 5 min) versus M-TraM. (C) Transcriptome (t = 15 min) versus M-TraM. (TIFF 376 kb) [file 1471-2164-14-299-S4.tiff]

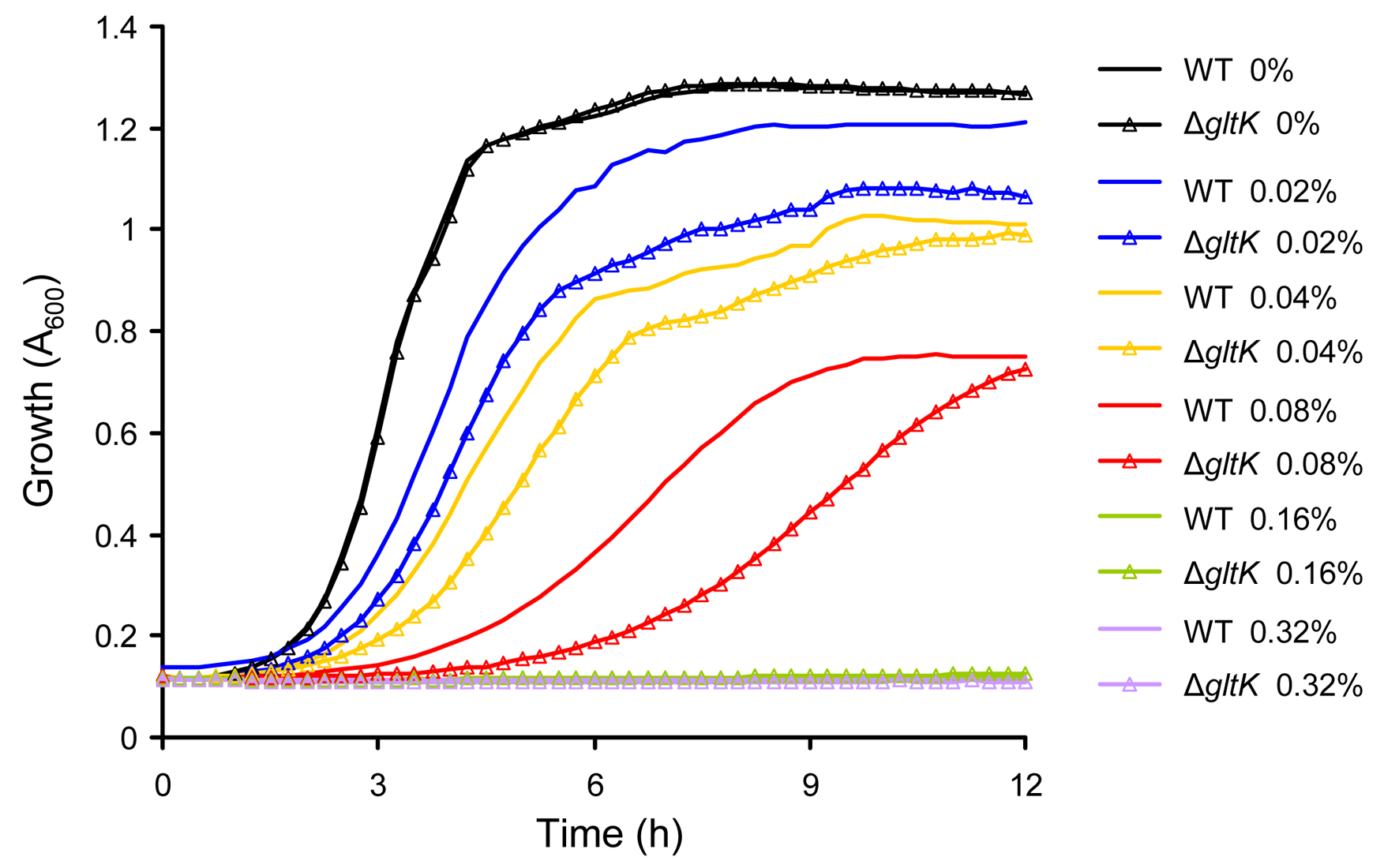

Supplement: Additional file 6: Figure S2 — Growth of E. faecium in BHI with different concentrations of bile salts. Overnight cultures of E. faecium strains were inoculated at an initial cell density of OD660 0.0025 in BHI or BHI with 0.02%, 0.04%, 0.08%, 0.16% and 0.32% of bile salts. Growth curves of wild-type E1162 and the ΔgltK mutant are shown. [file 1471-2164-14-299-S6.tiff]
